# Metabolite dependence of antibiotic susceptibility in a gut microbe

**DOI:** 10.1128/msphere.00600-24

**Published:** 2024-09-19

**Authors:** Kyle R. Allison

**Affiliations:** 1 Division of Infectious Diseases, Department of Medicine, Emory University School of Medicine, Atlanta, Georgia, USA; University of Nebraska Medical Center College of Medicine, Omaha, Nebraska, USA

**Keywords:** antibiotics, gut microbiota, metabolism, tolerance

## Abstract

Antibiotics save lives but can have unwanted effects on our gut microbes, thereby contributing to disease. A mechanistic understanding of how such microbes respond to antibiotics is hence critical. Recently in *mSphere*, Nilson et al. investigated the metabolite dependence of antibiotic susceptibility in *Bacteroides thetaiotaomicron*, an abundant and important member of our gut microbiota (R. Nilson, S. Penumutchu, F. S. Pagano, and P. Belenky, mSphere 9:e00103-24, 2024, https://doi.org/10.1128/msphere.00103-24). Their uncovered findings suggest the possibility of potentiating antibiotics with metabolites to reduce post-antibiotic “blooming” of *B. thetaiotaomicron* and the associated development of gut symbiosis.

## COMMENTARY

In 4 years, penicillin will turn 100. Actually, *Penicillium* molds have probably created penicillin for millions of years. But in 1928, one of these molds contaminated and killed Alexander Fleming’s *Staphylococcus aureus* culture (1). This was almost too fortuitous: Fleming was looking for ways to kill bacteria and previously discovered lysozyme by a similar “accident” (2). It can be difficult to appreciate, but cuts and minor wounds were routinely fatal because of bacterial infections. Fleming believed penicillin could change that (3), and it did.

Though more antimicrobials have been discovered and resistance has become widespread, β-lactams like penicillin remain the most-prescribed antibiotics. However, they can also alter our microbiome, i.e., the collection of microbes living on and in us. These microbes are not trivial; the gut microbiota has over 300 bacterial species: some good, some bad, and many somewhere in between. Increasingly, microbiota are implicated in medical conditions that have not been previously considered “microbial,” like obesity, inflammatory bowel disease, asthma, and cancer. (4). The conceptual shift under way is comparable to the 19th-century acceptance of “germ theory”; i.e., microbes produce infectious diseases. Effectively, it is germ theory, extended beyond infectious diseases. Together with evidence that antibiotics influence microbiota, this led to the “missing microbes” idea (5): broadly, microbiome disruptions contribute to modern increases in diverse diseases. β-Lactams have saved millions of people from infections, but they may have also driven other diseases.

Each microbe’s frequency in the microbiome—inside animals or patients, during diseases, or after perturbations—can be tracked by DNA sequencing without needing to culture them in the lab. Sequencing transformed microbiome research, but the evidence is often correlative rather than causal (4). Relatedly, germ theory took off when Robert Koch introduced criteria for proving a microbe caused a given disease. Koch’s postulates include isolating a microbe from infections, growing it in pure culture, and re-introducing it to healthy animals to produce infections. Focusing on the microbes themselves transformed medicine: inexplicable diseases became remarkably simple. Similar approaches to microbiome germs might similarly simplify some diseases, and seldom-studied “microbiome” bacteria are increasingly being studied in the lab.

*Bacteroides thetaiotaomicron* (with its salad of Greek letters theta, iota, and omicron) is an abundant and important gut microbe (6). It is a symbiotic, Gram-negative, anaerobe that breaks down polysaccharides in our food, providing nutrients to us and other microbes. *B. thetaiotaomicron* performs complicated roles in metabolism and immune response and can contribute to post-antibiotic infections. Often delivered orally, β-lactams can inhibit microbial growth in the gut. *B. thetaiotaomicron*, however, is tolerant and “blooms,” thereby increasing key metabolites for *Clostridium difficile* and *Salmonella enterica* (7)—pathogens that produce debilitating, dangerous, and difficult-to-treat infections. Intriguingly, metabolites can alter *B. thetaiotaomicron*’s β-lactam tolerance in mice (8). Hence, there might be a simple solution, via *B. thetaiotaomicron* metabolism, to prevent post-antibiotic gut infections, but the mechanism of tolerance was unclear.

Recently in *mSphere*, Rachel Nilson and colleagues from Peter Belenky’s lab investigated this mechanism (9). Rather than doing so in the complex environment of the gut, with its 300+ species, they studied *B. thetaiotaomicron*’s β-lactam susceptibility in pure cultures (Robert Koch would be pleased). Growing *B. thetaiotaomicron* on a range of different metabolites—including mono-, di-, and polysaccharides—they measured resistance to the β-lactam amoxicillin by minimum inhibitory concentration. Resistance was found to be up to 16-fold higher on the polysaccharides compared with simpler sugars, and consistent results were observed in the degree of bacterial killing by amoxicillin. β-Lactam susceptibility has been thought to correlate with bacterial growth rate (10) but not with *B. thetaiotaomicron*: the authors found that on polysaccharides, the microbes are less susceptible and grow slightly faster, suggesting gene regulatory control may be involved.

Living in the gut, *B. thetaiotaomicron* is likely accustomed to having an assortment of nutrients to choose from, and it is unclear what it might do when presented with both a simple (glucose) and complex (pectin) saccharide. Nilson et al. found that, regardless of pectin’s presence, sufficient glucose made cells susceptible. Metabolite-dependent susceptibility was observed with other β-lactams but interestingly not with the cephalosporin sub-class. This may indicate a rather complicated underlying mechanism that is worth future exploration. Relatedly, β-lactam resistance is often the result of β-lactamases, enzymes that inactivate the antibiotic, and *B. thetaiotaomicron* appears to have several. Nilson et al. present data indicating no detectable difference in β-lactamase production between conditions, and if anything, there may be increased β-lactamase activity when cells are grown on glucose. The data are suggestive but not yet conclusive regarding the involvement of the β-lactamases, and future confirmation might require inactivating or overexpressing them genetically.

To more comprehensively explore the underlying mechanism, Nilson et al. next profiled the transcriptional response of *B. thetaiotaomicron* to different nutrients and treatments. Perhaps unsurprisingly, growth on pectin induced genes for pectin degradation, a good sign that *B. thetaiotaomicron* knows what it is doing. On the other hand, these experiments also indicated several likely β-lactamases are upregulated on pectin, complicating the hypothesis on their lack of involvement. Additionally, several penicillin-binding proteins (PBPs; the β-lactams’ targets) were downregulated in pectin, which might also cause increased tolerance. From the perspective of susceptibility though, this indicates glucose might induce *B. thetaiotaomicron* to simultaneously drop two of its defenses to β-lactams, further suggesting its promise for potentiating antibiotics.

Parsing the transcriptional data, the authors note many global changes in metabolism genes between the glucose and pectin conditions. The role of metabolism in antibiotic susceptibility, while not a one-size-fits-all relationship, has been widely investigated (11). In fact, adding specific metabolites is likely the broadest and most effective available strategy to potentiate antibiotics. It has been applied to a variety of antibiotics, against a variety of bacteria, and uses metabolites that are generally non-toxic to patients. Nilson et al. thought stimulating the TCA cycle might further boost susceptibility, and it did: both in cultures with glucose or pectin, though only with specific metabolites (pyruvate and fumarate). This is an exciting result and further supports the idea that metabolites might be used to control antibiotic susceptibility in *B. thetaiotaomicron*. In the future, the underlying mechanisms will require further investigation: is it β-lactamases, PBPs, or metabolism that cause susceptibility? It might be all three of course. Does a global regulatory network, like carbon catabolite repression, control tolerance? If so, should we target it? However, remembering Koch, maybe it is time to return to the animals to see if the uncovered metabolite-dependent susceptibility can be used to reduce *B. thetaiotaomicron* blooming and prevent gut dysbiosis. There are many opportunities here.

An early demonstration of antibiotic potentiation by metabolites used previous mechanistic findings to improve already-existing aminoglycosides (12) and later was suggested as a way to mitigate the toxic side effects of these antibiotics (13). Nilson et al. and others are extending similar ideas beyond pathogens to address the unwanted side effects of antibiotics on our microbiota. Such microbe-focused mechanistic studies are needed to identify ways to mitigate harm to our microbiomes. In *B. thetaiotaomicron*, these efforts will be aided by rapid expansion and improvements in genetic techniques (14–17), though understanding the microbe’s behavior will also require observing the dynamics of individual cells. Fortunately, the finding that *B. thetaiotaomicron* can grow “nanaerobically” (very low oxygen) (18) has very cleverly been seized on to enable dynamic single-cell microscopy with green fluorescent protein (which needs oxygen for folding) (19). This opens experimental possibilities (20) regularly used in *Escherichia coli*, including investigation of its dynamic responses to antibiotics in individual cells (21, 22). Many microscope incubators enable oxygen control, suggesting that, for *B. thetaiotaomicron*, single-cell experiments might even become easier than bulk-scale experiments (which necessitates cumbersome anaerobic chambers). Our understanding of how antibiotics work requires such approaches, and perhaps the best way to focus on the microbes themselves is by adjusting the focus knob on a microscope.

Before antibiotics changed everything, they were only an idea. Paul Ehrlich, while working with Koch, identified dyes that stain microbes within patient samples. He reasoned he might find similar microbe-specific dyes that were also lethal. Such “magic bullets” would destroy infecting microbes and leave patients unharmed. Naturally, Ehrlich started infecting rabbits with syphilis and injected them with hundreds of dyes until their health improved. The resulting drug had toxicity issues, but he had proved the idea. Fleming took Ehrlich’s idea but Koch’s approach (while intentionally being untidy). He isolated pathogens and grew them in pure cultures, which he then left out on the chance something interesting might happen. In time, a killer mold landed in one of these cultures. As with fishing, which Fleming enjoyed (1), success resulted from knowing where to look and waiting patiently. In many ways, β-lactams remain the best embodiment of Ehrlich’s magical idea. While artificial intelligence is creating a renaissance in antibiotic discovery (23–25), our modern world exists because of our existing antibiotics. By protecting and improving them—through antibiotic stewardship, sensible rules for their use, potentiating them against pathogens, and mitigating harm to our microbiome—we just might prolong their magic, maybe even for hundreds of years to come.
